# ^68^Ga-PSMA Cerenkov luminescence imaging in primary prostate cancer: first-in-man series

**DOI:** 10.1007/s00259-020-04783-1

**Published:** 2020-04-02

**Authors:** Judith olde Heuvel, Berlinda J. de Wit-van der Veen, Henk G. van der Poel, Elise M. Bekers, Maarten R. Grootendorst, Kunal N. Vyas, Cornelis H. Slump, Marcel P. M. Stokkel

**Affiliations:** 1 Department of Nuclear Medicine, Netherlands Cancer Institute-Antoni van Leeuwenhoek, Amsterdam, The Netherlands; 2 Technical Medicine Center, University of Twente, Enschede, Netherlands; 3 Department of Urology, Netherlands Cancer Institute-Antoni van Leeuwenhoek, Amsterdam, Netherlands; 4 Department of Pathology, Netherlands Cancer Institute-Antoni van Leeuwenhoek, Amsterdam, Netherlands; 5 Lightpoint Medical Ltd., Chesham, UK

**Keywords:** Cerenkov imaging, 68-Gallium-PSMA intraoperative assessment, Positive surgical margin, Primary prostate cancer

## Abstract

**Purpose:**

Currently, approximately 11–38% of prostate cancer (PCa) patients undergoing radical prostatectomy have a positive surgical margin (PSM) on histopathology. Cerenkov luminescence imaging (CLI) using ^68^Ga-prostate-specific membrane antigen (^68^Ga-PSMA) is a novel technique for intraoperative margin assessment. The aim of this first-in-man study was to investigate the feasibility of intraoperative ^68^Ga-PSMA CLI*.* In this study, feasibility was defined as the ability to distinguish between a positive and negative surgical margin, imaging within 45 min and low radiation exposure to staff.

**Methods:**

Six patients were included in this ongoing study. Following perioperative i.v. injection of ~ 100 MBq ^68^Ga-PSMA, the prostate was excised and immediately imaged ex vivo. Different acquisition protocols were tested, and hotspots on CLI images from the intact prostate were marked for comparison with histopathology.

**Results:**

By using an acquisition protocol with 150 s exposure time, 8 × 8 binning and a 550 nm shortpass filter, PSMs and negative surgical margins (NSMs) were visually correctly identified on CLI in 3 of the 5 patients. Two patients had a hotspot on CLI from cancer < 0.1 mm from the excision margin.

**Conclusion:**

Overall, the study showed that ^68^Ga-PSMA CLI is a feasible and low-risk technique for intraoperative margin assessment in PCa. The remaining patients in this ongoing study will be used to assess the diagnostic accuracy of the technique.

Trial registration: NL8256 registered at www.trialregister.nl on 04/11/20109.

## Introduction

Approximately 11–38% of patients treated with radical prostatectomy have a positive surgical margin (PSM) on final histopathology [1, 2]. Although conflicting results on the long-term oncological effects on survival have been published, adjuvant local radiotherapy was found to reduce biochemical recurrence rate and is therefore offered to men with PSM [1, 3, 4]. Complete surgical excision is challenging as the surgeon currently depends on visual and tactile appearance to distinguish cancerous from normal tissue. Additional modalities for intraoperative margin assessment can enable more radical excision. NeuroSAFE is a histopathological technique that applies fresh-frozen section evaluation on areas near the neurovascular bundle during surgery. This technique is currently used in several clinics to reduce PSM rates while improving functional outcomes [5–7]. As this technique aims at conserving the neurovascular bundle, rather than actual margin assessment of the prostate surface, only the posterolateral part of the prostate will be sampled. Next, the NeuroSAFE technique is labour intensive, time consuming (~ 45 min) and prone to sampling errors as only a small part of the prostate is assessed [7, 8].

Other imaging-based technologies to assess margin status are being developed, one of which is Cerenkov luminescence imaging (CLI). Cerenkov radiation is produced when a charged particle (e.g., beta particle from isotopes such as fluorine-18, gallium-68, zirconium-89, yttrium-90) travels faster than the speed of light trough a dielectric medium such as tissue [9–11]. When the locally polarized medium along the path of that particle returns to its ground state, broad-spectrum electromagnetic radiation known as Cerenkov radiation is emitted. Because of the weak light intensity of Cerenkov radiation, it can only be detected by means of highly sensitive optical imaging systems in a dark environment. Roughly one decade ago, CLI became of interest to the biomedical field [12], after publications that modern sensitive CCD detectors are able to visualize Cerenkov radiation in mice. The initial studies using fluorine-18 and zirconium-89 showed that accurate visualization and quantification of Cerenkov radiation is possible [10–16]. The first clinical study of CLI was published in 2013 using Iodine-131 to image the thyroid gland [17], and in 2017, the feasibility of CLI for intraoperative margin assessment was demonstrated in breast cancer [18]. In the latter study patients received a preoperative injection of ^18^F-fluorodeoxyglucose (^18^F-FDG), followed by breast-conserving surgery and intraoperative imaging of excised wide local excision specimens in a dedicated CLI system to assess margin status.

Applicability of ^18^F-FDG in prostate cancer (PCa) is unfortunately limited, since there is a low uptake of glucose due to the low rate of glycolysis [19, 20]. With the introduction of tracers that target the prostate-specific membrane antigen (PSMA), the role of molecular imaging in PCa changed rapidly. Positron emission tomography (PET) using gallium-68 (^68^Ga) or ^18^F-labelled PSMA-ligands are increasingly used for primary staging in high-risk patients, with excellent diagnostic performance [21]. An important aspect for CLI is the initial positron energy of an isotope; the higher the initial energy, the longer the path length through tissue, and hence, the higher the Cerenkov yield. So, in addition to the receptor-targeted accumulation of PSMA-ligands in PCa lesions, the advantage of ^68^Ga-labelled radiopharmaceuticals for CLI is the high Cerenkov yield [16]. In our previous study, we demonstrated that the Cerenkov yield from ^68^Ga is approximately 22× higher compared to ^18^F, which should enable clinical translation of CLI in PCa [22].

The aim of this first-in-man clinical study was to assess the feasibility of ^68^Ga-PSMA CLI for intraoperative margin assessment in PCa. Here clinical feasibility of the technology is defined as the ability to distinguish between a PSM and a negative surgical margin (NSM) by imaging the entire prostate within 45 min (typical duration of the NeuroSAFE procedure) and with low radiation exposure to staff.

## Material and methods

### Patient inclusion

Present study was approved by the local Medical Ethics Committee (NL8256). Six high-risk primary PCa patients scheduled for robot-assisted radical prostatectomy (RARP) were enrolled in this ongoing trial after written informed consent was obtained. The main inclusion criteria were a tumour lesion > 1.5 cm on MRI, and a PSMA-positive intra-prostatic lesion on PSMA PET/CT. In all patients, CLI images were not used for surgical or histopathological decision-making.

### Surgery and intraoperative CLI

An intravenous injection of ~ 100 MBq [^68^Ga] Ga-HBED-CC-PSMA (ABX GmbH, Radeberg, Germany) was given immediately after the da Vinci® surgical system was docked. After removal, the prostate was prepared for Cerenkov imaging by rinsing it with sodium chloride to clear any potential radioactive contamination from blood or urine.

**Fig. 1:**
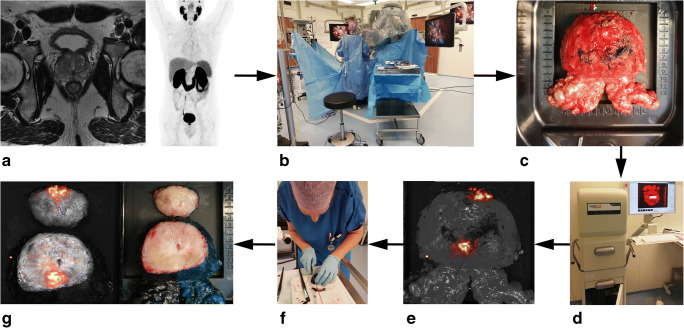
Workflow of the current CLI study, **a** A preoperative ^68^Ga-PSMA PET-CT and MRI scan are acquired 3–4 weeks prior to surgery as per standard of care, based on the result of these images patients are included. **b** During surgery ^68^Ga-PSMA is administered i.v. after the da Vinci® surgical system is docked. Radiation dose to all surgical staff is monitored. Note the position of the scrub nurse in close proximity to the patient. Once removed, the prostate is rinsed with NaCl to clear any radioactive urine and blood that could be present on the surface. **c** The prostate is positioned in a disposable specimen tray. **d** Images of all six sides of the prostate using the different settings are acquired in the CLI device. **e** Unfiltered Cerenkov image of the intact prostate specimen. **f** Upon image completion of the intact prostate, the prostate is inked and cleaved ~ 1 cm from the apex by a trained person. **g** Image of the cleaved prostate and the corresponding CLI image acquired with the same settings as the intact specimen to confirm tumour uptake and quantify the intensity in benign and cancerous tissue

Images from six sides of the prostate (left/right, anterior/posterior, basal/apical) were acquired using the LightPath® CLI system (Lightpoint Medical Ltd., Chesham, UK) (Fig. 1). This device consists of a light-tight specimen chamber, and acquires a photographic reference image and a Cerenkov image which are automatically overlaid [23]. To reduce the noise from high-energy gamma photons (called gamma strikes), the images are immediately processed by the LightPath® software by applying a Gaussian filter (σ = 3 pixels) and a median filter (3 × 3 pixels) and then displayed. In our previous in vitro study, it was found that images with 120 s exposure, 2 × 2 binning and no filter were sufficient for detection of clinically relevant ^68^Ga-PSMA activity levels [21]. This imaging protocol formed the basis for the current ex vivo clinical study. To account for potential differences in signal levels that can be expected when converting in vitr results to an ex vivo clinical setting, in the current study, images were acquired with different acquisition times (30, 60, 150, 300 s), pixel binning (2 × 2 [234 μm], 4 × 4 [469 μm], 8 × 8 [938 μm]) and optical filter (no filter and 550 nm shortpass filter) with the aim to identify an acquisition protocol that provided sufficient sensitivity within a time-window feasible for intraoperative use (< 45 min).

If areas of increased signal on CLI (called “hotspots”) were visualised on the intact images acquired without an optical filter, the acquisition was repeated with a 550 nm shortpass filter. As shorter-wavelength light has a higher attenuation and scattering in tissue, a persistent hotspot on the shortpass-filtered image indicates presence of PSMA-containing cancer cells near the excision margin. Based on visual assessment of the intact images, hotspots were marked with a suture for accurate comparison with histopathology.

Following CLI of the intact prostate specimen, the surface of the prostate was inked and cleaved 1 cm from the apex according to standard histopathological protocol. The cleaved prostate was subsequently imaged with CLI to visualize the primary lesion, to confirm presence of ^68^Ga-PSMA in the primary lesion and to quantify the signal in benign and cancerous tissue. The workflow of the CLI-procedure is displayed in Fig. 1.

### Radiation safety monitoring

As ^68^Ga-PSMA is not routinely used in the operating room, the radiation exposure to the operating room staff was monitored using electronic personal radiation dosimeters (MGP Instruments DMC 2000; Mirion Technologies, Ltd.). Measurements included the scrub nurse positioned directly next to the patient, the surgeon positioned behind the da Vinci surgical robot at ~ 3 m from the patient, and the anaesthetist, periphery nurse and researcher positioned more than 1 m away from the patient.

### Histopathology

After completion of CLI, the specimens were taken to the pathology department. Histopathological examination was performed as per standard of care by an experienced Uropathologist (EMB). A PSM was defined as cancer extending into the inked surface [24].

### Image analysis

**Fig. 2:**
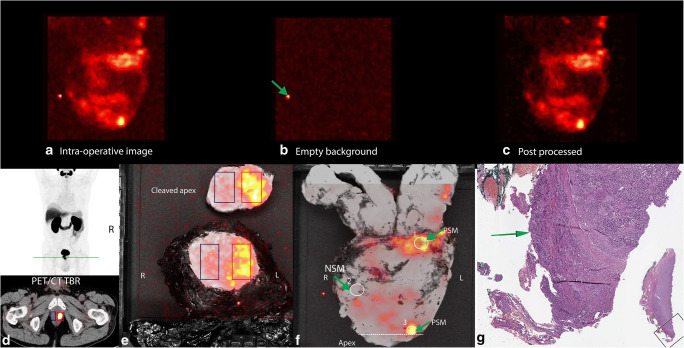
Overview of the image analysis and quantification methods. **a** Intraoperative Cerenkov image of the specimen after median and Gaussian filtering. This image is used for visual identification of PSMs and NSMs. **b** A Cerenkov image of an empty background. Note the presence of the defective pixel (green arrow). **c** Post-processed Cerenkov image after background subtraction. Note that the background subtraction removes the defective pixel on the post-processed image. **d** The PSMA PET/CT scan. The green line on the maximum intensity profile (MIP) image indicates the location where the prostate is cleaved. The transverse image of the fused PET/CT scan highlights the location of the tumour (red ROI) and benign tissue (blue ROI). **e** Intraoperative CLI image of the prostate from patient 2 after the specimen was cleaved at the apex, showing the tumour (red ROI) and the benign tissue (blue ROI). **f** CLI image of intact prostate specimen from patient 2. Two hotspots, corresponding with a histopathological PSM, can be identified (ROI 1 and ROI 3). Areas with no increased signal, corresponding with benign tissue from a NSM, can also be seen (ROI 2). For clarity, each ROI is also highlighted with a green arrow. For this patient the TBR was calculated by dividing the radiance from ROI 1 and ROI in 2. The dotted white line represents the location where the prostate was cleaved. **g** Histopathology image of the area corresponding with ROI 3 on the intact CLI image. A PSM can be identified (green arrow)

In addition to visual assessment of the intact prostate image to qualify if the margins are positive or negative, explorative quantitative assessment was performed after surgery using MATLAB R2017b (The MathWorks, Natrick, WA). The researcher (JoH) who performed this post hoc evaluation was blinded to the histopathology results, but had access to the patient’s PSMA-PET/CT and MRI. First, the images were post-processed by subtracting a background image (i.e. a CLI image of an empty specimen tray acquired with the same acquisition settings) from the clinical CLI images. This was performed to remove any camera noise in the image as well as the artefact from a defect pixel in the camera (Fig. 2). Measurements of the mean radiance (photons (p)/s/cm^2^/sr) were performed by manually selecting regions of interest (ROI) on the post-processed images of both the intact and cleaved prostate images around areas showing hotspots (tumour) and in areas with no increased signal (tissue background), and based on the latter ROIs, the tumour-to-background ratios (TBRs) were calculated. The quantitative analysis of the cleaved prostate images was performed with the aim to eventually identify a quantitative threshold for discriminating between a PSM and NSM. To enable comparison of signal levels between images of each patient individually, the signals (tissue background and tumour) were corrected for radioactive decay. Additionally, decay-corrected radiances were also corrected for injected activity to enable comparison of images between patients (corrected radiance). The process of image analysis is displayed in Fig. 2.

## Results

Six patients were initially included into the study, but one patient had to be excluded due to unsuccessful ^68^Ga-PSMA labelling on the day of surgery. The mean injected activity was 83 MBq ± 19, and the mean time between injection and CLI acquisition of the prostate was 73 ± 14 min. An overview of the patient characteristics, ^68^Ga-PSMA activity, CLI results and histopathological results can be found in Table 1. Two out of five patients had a PSM according to histopathology (patient 1 and 2).

**Table 1:** Patient characteristics and CLI and histology results of all 5 patients

	Patient 1	Patient 2	Patient 3	Patient 4	Patient 5
Age (y)	67	71	58	73	63
Weight (kg)	75	75	106	126	86
Preoperative tumour grade	cT3bN0M0	cT1cN0M0	cT1cN0M0	cT1cN0M0	cT2cN0M0
Postoperative tumour grade	pT3aN0Mx	pT3aN0Mx	pT2cN0Mx	pT3bN1Mx	pT2N0Mx
iPSA (μg/L)	29.9	4.4	5.3	8.3	6.4
Gleason Score	4 + 4 = 8	4 + 4 = 8	4 + 4 = 8	4 + 5 = 9	4 + 9
Prostate volume MRI (cc)	47	30	41	76	28
SUV_max_ on PSMA PET/CT	24.8	31.6	4.3	49.2	21.2
Injected ^68^Ga-PSMA activity (MBq)	118	68	88	76	65
Activity at start of CLI (MBq)	45	51	38	42	34
Suspected PSM CLI	Yes	Yes	No	Yes	Yes
PSM on histopathology	Yes	Yes	No	No	No
Location agreement CLI – Hpath	Yes	Yes	N/A	Yes**	Yes**
Closeness tumour to surface (mm)	0	0	3	0.1	0.1
Corrected radiance hotspot intact prostate (p/s/cm^2^/sr) 550 nm filter/no filter	1497/x	1446/16691 1711/14533	798/11139	1617/16474	1264/12500
TBR intact prostate 550 nm filter/no filter	3.3/x	4.0/4.7 4.7/4.1	2.5/2.5	3.3/2.7	7.7/2.5
Corrected radiance hotspot cleaved prostate (p/s/cm^2^/sr) 550 nm filter/no filter	2135/x	1149/12554	^	1783/16159	2642/18430
TBR cleaved prostate 550 nm filter/no filter	3.1/x	2.2/1.8	^	3.1/2.6	5.9/1.8

The displayed radiances are activity and decay corrected. *iPSA*, initial prostate specimen antigen level; *HPath*, histopathology; *TBR*, tumour-to-background ratio. ^In this patient, the tumour was located in the base of the prostate, while the prostate was cleaved ~1 cm from the apex, thus preventing visualization of the tumour on CLI. **Although no histopathological PSM, the location of the hotspot on CLI agreed to a tumour to ink distance of < 0.1 mm on the histopathology slide. *x*, No non-filtered image taken. *N/A*, As there was a NSM in patient 3,4,5, there was no TBR calculated from the intact specimen. Note that patient 2 had a PSM on two locations; therefore the corrected radiance of both hotspots is displayed, as well as the TBR of both locations on the intact prostate. Pre- and postoperative tumour grade are based on the clinical TNM stage [26]: *T* for primary tumour, *N* for nodal stage, *M* for metastases. *R* stands for margin status and *X* indicates that the status of a certain characteristic cannot be determined. *c* for clinical, which indicates that the classification is based on clinical parameters. *p* for pathological, which indicates that the classification was performed in this case on the excised prostate in the pathology lab

**Fig. 3:**
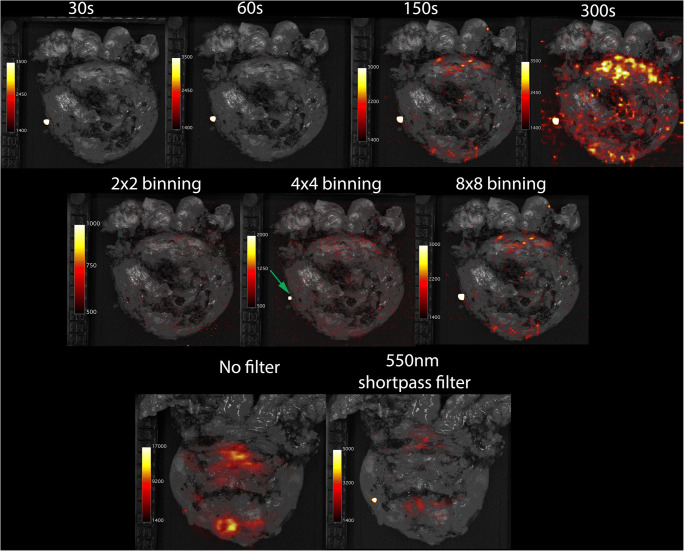
Results of protocol optimisation process with different acquisition settings. The images in the **upper row** are from patient 1 who had a histopathological PSM at the apex. All images were acquired with 8 × 8 pixel binning and 550 nm shortpass filter but with different exposure times of 30 s (TBR:1.10), 60 s (TBR:1.18), 150 s (TBR:1.85) and 300 s (TBR:1.98). The images in the **centre row** are also from patient 1and were acquired with 150 s exposure time and filter, but different pixel binning 2 × 2 (TBR:1.06), 4 × 4 (TBR:1.26) and 8 × 8 (TBR:1.85). The **bottom row** images are from patient 3 and were acquired with 150 s exposure time and 8 × 8 binning, but without a filter (TBR:4.33) and with filter (TBR:1.85), respectively. Note that the area of increased signal with the green arrow appears in all non-background subtracted images; this is an artefact from a defect pixel in the camera of the CLI system, and can be ignored. The colour bar indicates the scale of counts

**Fig. 4:**
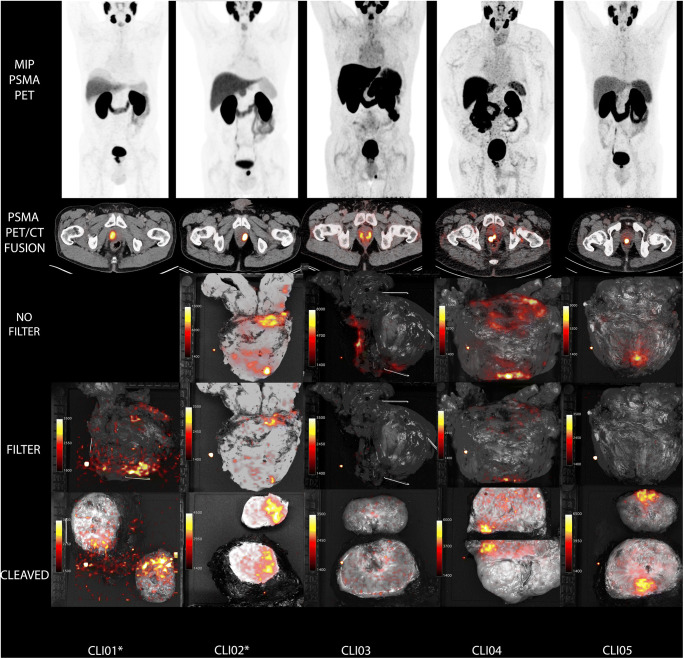
An overview of the PET/CT scans and CLI images of all five patients. **a** The MIP of the PSMA PET-scan. Note that these scans were obtained using ^68^Ga-HBEDCC-PSMA (patient 5) and ^18^F-DCFPyl (patient 1, 2, 3, 4), hence the difference in PSMA-tracer distribution. **b** The transverse image of the fused PSMA PET/CT-scan at the level of the prostate lesion. **c** Non-filtered CLI images of intact prostate specimen. Note that patient one did not have a non-filtered image taken. **d** Filtered CLI images of intact prostate specimen. **e** Non-filtered CLI images of the cleaved prostate. The prostate specimen was cleaved at ~ 1 cm from the apex. Note that in patient 3, it was not located in the apex region but in the base, and therefore no tumour signal is visible on the CLI image. Scaling of the PET-scans was performed based on the intensity of the liver; CLI images were scaled visually based on the benign background signal. The displayed CLI images are the intraoperative images that are not corrected for background which explains the presence of the defective pixel. All CLI images were acquired with 150 s exposure time and 8 × 8 binning. *The patient numbers marked with a star had a PSM based on histopathology

Figure 3 displays images with variable binning, filters and exposure times, which were used to evaluate and optimize the CLI acquisition settings on the intact prostate. An exposure time of 150 s was sufficient to detect cancerous lesions on CLI on both the intact prostate specimen (Figs. 3 and 4. Based on the intact prostate images, a 150-s exposure was favoured over a 300-s acquisition as the TBR for the latter did not sufficiently improve to outweigh the increase in acquisition time (TBR 1.85 vs 1.98). An 8 × 8 pixel binning improved the TBR compared with 2 × 2 and 4 × 4 binning, respectively (TBR 1.85, 1.26, and 1.06). Acquisitions without a filter had a higher TBR (4.33) compared to the ones with filter (TBR 1.85).

Data from the CLI images of the cleaved prostate showed that the tumour had a higher radiance as compared with the benign tissue, both on the filtered (mean TBR 3.6 ± 1.4) and non-filtered images (mean TBR 2.1 ± 0.4), Table 1. As expected, the use of a filter decreased the radiance in both tumour and tissue background areas. In patient 3, the tumour could not be identified on the cleaved prostate image, as the tumour was not located in the apex region where the incision was made but in the base.

In the two patients with a PSM (patient 1 and 2), hotspots were identified in corresponding areas on both the non-filtered and the filtered intact prostate image, thus enabling successful identification of PSM on CLI (Fig. 4). Three out of five patients had a histopathological NSM. In one of these patients (patient 3), the NSM was correctly identified on CLI as the hotspot was present on the non-filtered images but not on the filtered image, potentially indicating PSMA-containing cells further away from the resection margin (Fig. 4). In the other two patients (patient 4 and 5), a hotspot was identified on the non-filtered and in one patient as well on the filtered CLI image, and in both cases, histopathology found tumour cells < 0.1 mm from the margin. In patient 5, the hotspot was not clearly visible on the filtered intact image; however, the quantitative TBR was high at that location.

The sterile scrub nurse received the highest dose because of their close proximity to the patient: 0.016 mSv. The average dose to the anaesthetist, surgeon, periphery nurse and researcher were substantially lower: 0.001 mSv, 0.005 mSv, 0.002 mSv and 0.001 mSv, respectively.

## Discussion

In the current study, we present the first-in-man usage of intraoperative ^68^Ga-PSMA CLI in primary PCa. The results from the first patients enabled optimisation of the imaging protocol, and demonstrated the technical ability to correctly identify PSMs. The use of optical filters improves the ability to visually distinguish between PSM and NSM on CLI, a finding that has not been reported before. Importantly, the performed workflow was considered clinically feasible and safe for surgical staff in terms of radiation exposure levels.

These initial results show that by using an administered activity of ≥ 65 MBq ^68^Ga-PSMA, a 150-s acquisition time with 8 × 8 pixel binning, and specimen imaging with and without 550 nm shortpass filter, a PSM can be successfully detected. Compared with the published ^18^F-FDG CLI results in breast cancer, the current protocol uses a 3 times lower activity and a 2 times shorter acquisition time which is possible due to the higher tracer uptake and Cerenkov yield of ^68^Ga-PSMA [18]. Despite the longer duration of prostate cancer surgery, the lower injected activity and shorter half-life resulted in a roughly 2 times lower staff exposure. The radiation exposure to personnel, a maximum of 0.016 mSv per procedure, was within the International Commission on Radiological Protection limits [27]. Based on these values, a single scrub nurse can perform a minimum of 62 CLI procedures before exceeding the limits; a surgeon can perform 200 CLI procedures. All 6 sides of the prostate could be assessed with the CLI within approximately 20 min, which is well within the time window acceptable for intraoperative use. Although CLI may still delay surgery to some extent, the procedure is approximately twice as fast than a typical NeuroSAFE procedure that is only performed on the posterolateral surface of the prostate. Furthermore, CLI enables whole-specimen assessment in the operating theatre without the need for a dedicated pathology department, contrarily to NeuroSAFE. Next, when a lymph node dissection is performed as well, the surgery can continue with the dissection while CLI imaging of the prostate occurs.

In 3 out of 5 patients, the histopathological PSMs and NSMs were correctly detected on CLI. In two patients, a hotspot on the Cerenkov image from the intact prostate was obtained from a tumour deposit at < 0.1 mm from the inked surface. These false-positive results can be explained by the physical properties of ^68^Ga. The CLI signal is not produced in one spatial location, but instead along the positron trajectory in tissue (± 2.8 mm on average for ^68^Ga, which is the same as the PET range) [28]. Although the use of an optical filter restricts the detected signal to enable visualisation of activity on the surface [29], it remains hard to quantify from what depth this activity actually originates. As identified in this first-in-man study, depth estimation is a key challenge of CLI and an important limitation compared with NeuroSAFE. The spatial accuracy that is achieved with histopathological evaluation is far greater than will ever be achieved using CLI. This aspect is further accomplished by the fact that it is difficult to estimate the real amount of activity present in the prostate after administration based on the Cerenkov images alone. In the future, the amount of activity present in the excised prostate specimen could be measured using dose calibrators.

A quantitative approach could help to discriminate between benign and tumour based on a threshold value, rather than on visual interpretation alone, since visual assessment is inherently prone to subjective window-level settings, and may result in erroneous identification of hotspots. In this study, each prostate was cleaved to enable visualisation and quantification of benign and cancerous tissue in the apex. This resulted in a mean TBR > 2, showing that the technology is able to sufficiently differentiate between tumour and benign tissue. Still, the process of cleaving is not preferred in the eventual work up, as it is time intensive and requires additional training. Our ongoing study will explore the possibility to define a TBR based on preoperative PET/CT-scans to recognize and to quantify potential areas that have a higher risk for PSMs on Cerenkov images based on radiance values. However, in the current feasibility study, the numbers were too small to make quantitative conclusions from the radiance levels. For quantification, the time between injection and imaging, administered activity, can be used to normalise the data. Additional variables that influence the CLI signal that are more challenging to account for are the PSMA expression of the tumour, and the fact that CLI uses optical imaging which means that scattering and attenuation from superficial tissue and blood can alter the Cerenkov signal intensity.

Though the current histopathological definition of a PSM is 0 mm (i.e. tumour on ink) [24], it is suggested that CLI could still provide valuable information on margin status, not only to the surgeon but also to inform the pathologist about areas at risk to guide more detailed histopathological evaluation. Another possibility is to guide the NeuroSAFE procedure itself by indicating suspicious areas, so that only those areas could be sampled for NeuroSAFE assessment, thereby reducing the risk of sampling errors and the duration of the NeuroSAFE procedure.

The current feasibility study has several limitations. Firstly, this feasibility study only addresses a small selective population. The patients were selected based on a high chance of a PSM, thus not representative for the general population that will undergo RARP. Still, the included patients are the ones that could benefit the most from intraoperative margin assessment [29]. Secondly, the administered activity varied between the patients. The ^68^Ga-PSMA was produced locally at specific time slots each day due to the fact that this is a generator product. Thus, the actual time of surgery needs to correspond to the ^68^Ga-PSMA production time. Batches for multiple patients are retrieved from one production; therefore, once the syringe is prepared for the study patient, and surgery is delayed, the activity cannot be adjusted accordingly. This is further complicated by the relatively short half-life of ^68^Ga (68 min). To account for lower ^68^Ga-PSMA activity levels due to delays in the start of surgery, the exposure time could be increased, thereby increasing TBR and ultimately aiding the visual identification of PSM on CLI. Still, variation in activity present in the prostate is normal considering a different PSMA expression between patients, and even within one prostatic lesion. This could be correlated to the SUV from preoperative PET/CT scans.

## Conclusion

This first-in-man study demonstrates that ^68^Ga-PSMA CLI is a promising and safe technique for intraoperative margin assessment in PCa. The validated acquisition settings enable detection of PSMs within an acceptable time window for intraoperative use, and acceptable radiation exposure to staff. The ongoing trial will further evaluate the diagnostic accuracy of CLI in a larger population and will assess the ability to establish a quantitative threshold to discriminate PSM from NSM, in addition to visual inspection alone.

## References

[1] Yossepowitch O, Briganti A, Eastham JA, Epstein J, Graefen M, Montironi R (2014). Positive surgical margins after radical prostatectomy: a systematic review and contemporary update. Eur Urol.

[2] Stephenson AJ, Wood DP, Kattan MW, Klein EA, Scardino PT, Eastham JA (2009). Location, extent and number of positive surgical margins do not improve accuracy of predicting prostate Cancer recurrence after radical prostatectomy. J Urol.

[3] Silberstein J, Eastham J (2014). Significance and management of positive surgical margins at the time of radical prostatectomy. Indian J Urol.

[4] Bolla M, van Poppel H, Tombal B, Vekemans K, Da Pozzo L, de Reijke TM (2012). Postoperative radiotherapy after radical prostatectomy for high-risk prostate cancer: long-term results of a randomised controlled trial (EORTC trial 22911). Lancet.

[5] Schlomm T, Tennstedt P, Huxhold C, Steuber T, Salomon G, Michl U (2012). Neurovascular structure-adjacent frozen-section examination (NeuroSAFE) increases nerve-sparing frequency and reduces positive surgical margins in open and robot-assisted laparoscopic radical prostatectomy: experience after 11,069 consecutive patients. Eur Urol.

[6] Mirmilstein G, Rai BP, Gbolahan O, Srirangam V, Narula A, Agarwal S (2018). The neurovascular structure-adjacent frozen-section examination (NeuroSAFE) approach to nerve sparing in robot-assisted laparoscopic radical prostatectomy in a British setting – a prospective observational comparative study. BJU Int.

[7] Beyer B, Schlomm T, Tennstedt P, Boehm K, Adam M, Schiffmann J (2014). A feasible and time-efficient adaptation of NeuroSAFE for da Vinci robot-assisted radical prostatectomy. Eur Urol.

[8] Dinneen EP, Van Der Slot M, Adasonla K, Tan J, Grierson J, Haider A, et al. Intraoperative Frozen Section for Margin Evaluation During Radical Prostatectomy: A Systematic Review. Eur Urol Focus. 2019:1–10. 10.1016/j.euf.2019.11.009 31787570

[9] Spinelli AE, D’Ambrosio D, Calderan L, Marengo M, Sbarbati A, Boschi F (2010). Cerenkov radiation allows in vivo optical imaging of positron emitting radiotracers. Phys Med Biol.

[10] Robertson R, Germanos MS, Li C, Mitchell GS, Cherry SR, Silva MD (2009). Optical imaging of Cerenkov light generation from positron-emitting radiotracers. Phys Med Biol.

[11] Ruggiero A, Holland JP, Lewis JS, Grimm J (2010). Cerenkov luminescence imaging of medical isotopes. J Nucl Med.

[12] Ciarrocchi E, Belcari N (2017). Cerenkov luminescence imaging: physics principles and potential applications in biomedical sciences. EJNMMI Phys.

[13] Beattie BJ, Thorek DLJ, Schmidtlein CR, Pentlow KS, Humm JL, Hielscher AH. Quantitative Modeling of Cerenkov Light Production Efficiency from Medical Radionuclides. Gelovani JG, editor. PLoS One. 2012;7:e31402. 10.1371/journal.pone.0031402 PMC3282695 22363636

[14] Holland JP, Normand G, Ruggiero A, Lewis JS, Grimm J (2011). Intraoperative imaging of positron emission tomographic radiotracers using Cerenkov luminescence emissions. Mol Imaging.

[15] Thorek DLJ, Ogirala A, Beattie BJ, Grimm J (2013). Quantitative imaging of disease signatures through radioactive decay signal conversion. Nat Med.

[16] Gill RK, Mitchell GS, Cherry SR (2015). Computed Cerenkov luminescence yields for radionuclides used in biology and medicine. Phys Med Biol.

[17] Spinelli AE, Ferdeghini M, Cavedon C, Zivelonghi E, Calandrino R, Fenzi A (2013). First human Cerenkography. J Biomed Opt.

[18] Grootendorst MR, Cariati M, Pinder SE, Kothari A, Douek M, Kovacs T (2017). Intraoperative assessment of tumor resection margins in breast-conserving surgery using 18 F-FDG Cerenkov luminescence imaging: a first-in-human feasibility study. J Nucl Med.

[19] Liu Y (2014). Diagnostic role of fluorodeoxyglucose positron emission tomography-computed tomography in prostate cancer. Oncol Lett.

[20] Jadvar H (2016). Is there use for FDG-PET in prostate cancer?. Semin Nucl Med.

[21] Corfield J, Perera M, Bolton D, Lawrentschuk N. 68Ga-prostate specific membrane antigen (PSMA) positron emission tomography (PET) for primary staging of high-risk prostate cancer: a systematic review. World J Urol. 2018:519–27. 10.1007/s00345-018-2182-1 29344682

[22] olde Heuvel J, de Wit-van der Veen BJ, Vyas KN, Tuch DS, Grootendorst MR, Stokkel MPM (2019). Performance evaluation of Cerenkov luminescence imaging: a comparison of 68Ga with 18F. EJNMMI Physics.

[23] Ciarrocchi E, Vanhove C, Descamps B, De Lombaerde S, Vandenberghe S, Belcari N (2018). Performance evaluation of the LightPath imaging system for intra-operative Cerenkov luminescence imaging. Phys Medica.

[24] Tan PH, Cheng L, Srigley JR, Griffiths D, Humphrey PA, Van Der Kwast TH (2011). International Society of Urological Pathology (ISUP) consensus conference on handling and staging of radical prostatectomy specimens. Working group 5: surgical margins. Mod Pathol.

[25] Buyyounouski MK, Choyke PL, McKenney JK, Sartor O, Sandler HM, Amin MB, et al. Prostate cancer - major changes in the American Joint Committee on Cancer eighth edition cancer staging manual. CA Cancer J Clin. 2017;67:245–53. 10.3322/caac.21391 PMC6375094 28222223

[26] International Commission on Radiological Protection (ICRP). The 2007 Recommendations of the International Commission on Radiological Protection. ICRP publication 103. Ann. ICRP. 2007;37:1–332. 10.1016/j.icrp.2007.10.003 18082557

[27] Moses WW (2011). Fundamental limits of spatial resolution in PET. Nucl Instrum Methods Phys Res Sect A.

[28] Sandell J, Zhu T (2013). A review of in-vivo optical properties of human tissues and its impact on PDT. J Biophotonics.

[29] Wieder JA, Soloway MS (1998). Incidence, etiology, location, prevention and treatment of positive surgical margins after radical prostatectomy for prostate cancer. J Urol.

